# Heterotypic vaccination responses against SARS-CoV-2 Omicron BA.2

**DOI:** 10.1038/s41421-022-00435-w

**Published:** 2022-07-19

**Authors:** Zhenhao Fang, Lei Peng, Carolina Lucas, Qianqian Lin, Liqun Zhou, Luojia Yang, Yanzhi Feng, Ping Ren, Paul A. Renauer, Valter S. Monteiro, Anne M. Hahn, Jonathan J. Park, Xiaoyu Zhou, Yale SARS-CoV-2 Genomic Surveillance Initiative, Kendall Billig, Mallery I. Breban, Christopher Castaldi, Chrispin Chaguza, Nicholas Chen, David Ferguson, Nicholas Kerantzas, Tobias R. Koch, Bony De Kumar, Marie L. Landry, David Peaper, Kien Pham, Wade Schulz, Irina R. Tikhonova, Chantal B. F. Vogels, Nathan D. Grubaugh, Craig B. Wilen, Sidi Chen

**Affiliations:** 1 Department of Genetics, Yale University School of Medicine, New Haven, CT USA; 2 System Biology Institute, Yale University, West Haven, CT USA; 3 Center for Cancer Systems Biology, Yale University, West Haven, CT USA; 4 Department of Immunobiology, Yale University, New Haven, CT USA; 5 Immunobiology Program, Yale University, New Haven, CT USA; 6 Molecular Cell Biology, Genetics, and Development Program, Yale University, New Haven, CT USA; 7 Department of Epidemiology of Microbial Diseases, Yale School of Public Health, New Haven, CT USA; 8 Department of Ecology and Evolutionary Biology, Yale University, New Haven, CT USA; 9 MD-PhD Program, Yale University, New Haven, CT USA; 10 Department of Laboratory Medicine, Yale University, New Haven, CT USA; 11 Comprehensive Cancer Center, Yale University School of Medicine, New Haven, CT USA; 12 Stem Cell Center, Yale University School of Medicine, New Haven, CT USA; 13 Center for Biomedical Data Science, Yale University School of Medicine, New Haven, CT USA; 14 Yale School of Public Health, New Haven, CT USA; 15 Yale Center for Genome Analysis, New Haven, CT USA; 16 Yale School of Medicine, New Haven, CT USA

**Keywords:** Immunology, Biological techniques

Dear Editor,

Coronavirus disease 2019 (COVID-19) pandemic has caused over 6 million deaths in the past 2 years, and continues to pose a significant threat to the world due to the increased transmissibility, infectivity, and immune evasion of continuously emerging variants^1^. Within weeks since its first identification, the newly emerged variant of concern (VOC), Omicron (lineage B.1.1.529) became the dominant variant and spread rapidly worldwide^2^. From the initial spread of Omicron, B.1.1.529 emerged two sub-lineages, BA.1 (alias of B.1.1.529.1) and BA.2 (alias of B.1.1.529.2). After Omicron BA.1 became globally dominant, it was rapidly displaced in most regions by BA.2^2^, leading the World Health Organization to classify Omicron BA.2 as another VOC^3^. The on-going COVID-19 “waves” are predominantly associated with BA.2 and BA.2.12.1.

Compared to the ancestral/wild-type (WT) virus, Omicron variants contain a high number of mutations in spike protein, which is the primary target of clinical antibodies and vaccines. The substantial differences between WT and Omicron spikes lead to extensive immune escape of Omicron from WT mRNA vaccine^4^, which prompted the idea of developing Omicron-specific vaccines. We generated several COVID-19 variant-specific mRNA vaccine candidates (including Omicron BA.1)^5,6^ which were designed based on variants’ spike stabilized by six proline mutations^7^. Variant-specific vaccine candidates, or lipid nanoparticle (LNP)-mRNAs, unequivocally exhibited advantage over WT LNP-mRNA in terms of eliciting neutralizing antibody against cognate antigens^5,6^. Moreover, immune profiling of Omicron BA.1 LNP-mRNA showed a significant boosting effect on waning immunity of WT LNP-mRNA-vaccinated mice to both Delta and Omicron BA.1 variants.

Omicron BA.1 and BA.2 share 21 mutations, but differ in 25 sites (Fig. 1a, b). Because of this antigenic divergence, Iketani et al. showed that BA.2 exhibited differential resistance profile to monoclonal antibodies than BA.1^8^. It is worth noting that BA.2 showed remarkable resistance to all clinical monoclonal antibodies except for recently authorized bebtelovimab^8^. The significant difference of BA.1 and BA.2 spikes raises a number of important questions. For instance, how potent is the immunity elicited by heterotypic vaccination, with WT or variant-specific LNP-mRNAs, against BA.2? How does this immune response compare to the response to BA.1? Does heterologous boosting with BA.1 LNP-mRNA or homologous boosting with WT LNP-mRNA remain effective against BA.2?

**Fig. 1:**
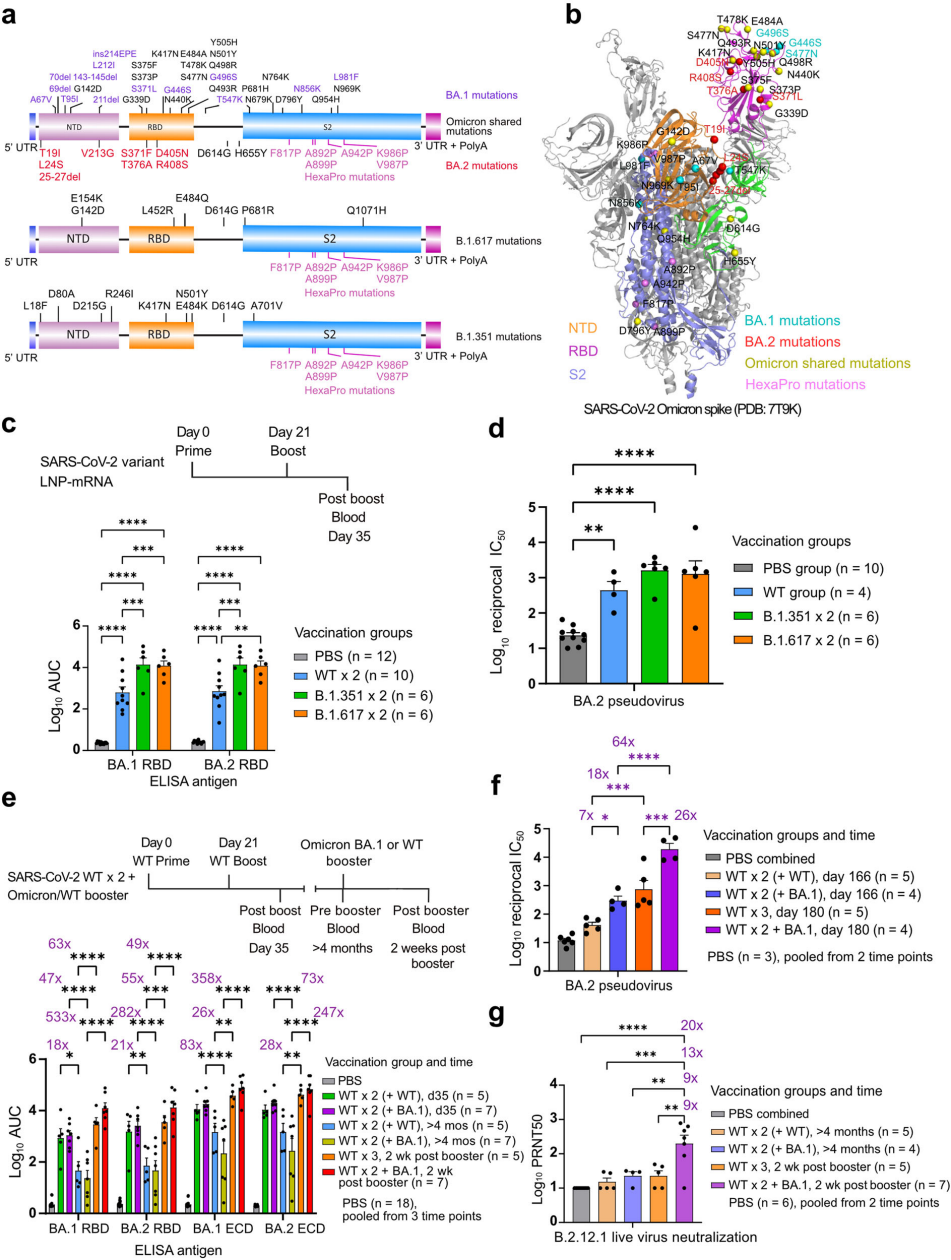
WT and variant-specific LNP-mRNA elicited potent antibody response against Omicron BA.1 and BA.2. Schematics (**a**) and spike structure (**b**) showing Omicron BA.1 and BA.2 mutations distribution in the vaccine design (a) and one protomer of BA.1 spike trimer (b, PDB: 7T9K). **c** Comparison of antibody response induced by two doses of 1 µg WT, B.1.351 or B.1.617 LNP-mRNA at 21 days interval. Antibody titers were determined by area under curve (AUC) of ELISA titration curves in Supplementary Fig. S1. **d** Neutralization of Omicron BA.2 pseudovirus by serum samples from mice vaccinated with 1 µg WT, B.1.351 or B.1.617 LNP-mRNA as illustrated in **c**. The neutralizing titers were quantified by log_10_ reciprocal IC_50_ and calculated from titrations in Supplementary Fig. S2. Gating strategy to select GFP positive infected cells is shown in Supplementary Fig. S5. **e** BA.1 and WT boosters strengthened waning immunity against Omicron BA.1 and BA.2 variants. ELISA antibody titers of samples from mice sequentially vaccinated with two doses of 1 µg WT LNP-mRNA followed by 10 µg WT (WT × 3, *n* = 5) or Omicron BA.1 (WT × 2 + BA.1, *n* = 7) LNP-mRNA boosters. The pre-booster groups (day 35 and > 4 month from prime) to receive WT or BA.1 boosters were denoted as WT × 2 (+ WT) and WT × 2 (+ BA.1) respectively. **f** Neutralization of Omicron BA.2 pseudovirus by plasma samples from mice in **e**. **g** Plaque-reduction neutralization (PRNT50) of Omicron BA.2.12.1 infectious virus by plasma samples from mice in **e**. Related statistical information and all source data can be found in Supplementary Information or Supplementary Dataset S1. Data points of PBS group showed no statistical difference between collection time points and were combined to one group in **e**–**g**. Only comparisons between adjacent time points or groups of same time point were shown in **e**, **f**. A subset of collected samples were characterized in pseudovirus and infectious virus neutralization assay due to depletion of samples.

To answer these questions, we characterized the antibody response induced by WT or variant specific LNP-mRNAs to Omicron BA.2 and compared it with immune response to BA.1. Samples used for BA.2 characterization were collected from mice that received two doses of 1 µg WT, B.1.351 (Beta variant) or B.1.617 (ancestor to the Delta variant (lineage B.1.617.2)) LNP-mRNAs^6^. All three LNP-mRNAs elicited significant antibody response to BA.2 (Fig. 1c, d; Supplementary Figs. S1, S2). Both B.1.351 and B.1.617 LNP-mRNA groups showed a trend of higher binding and neutralizing titers than WT group. Because of the strong selection pressure in the spike protein, emerging variants often share key mutations (i.e., convergent evolution)^9^. BA.2 shares 3 spike mutations with B.1.351 (K417N, N501Y, D614G) and B.1.617 (G142D, D614G, P681R), which may explain why the antibody response to BA.2 was higher in these two variants LNP-mRNA groups compared to WT (Fig. 1a). In all three vaccination groups, antibody response to BA.2 was similar to that of BA.1 (Fig. 1c), suggesting approximately equal reactivity of BA.1 and BA.2 to vaccination by WT, B.1.351 and B.1.617 LNP-mRNAs. It is worth noting that both BA.1 and BA.2 share the same three mutations with B.1.351 and B.1.617, which contributed to the conserved cross reactivity to two Omicron sublineages.

Given the BA.2 neutralization titer advantage of variant LNP-mRNA over WT, we went on to profile the antibody response of BA.1 LNP-mRNA to BA.2. To model the real-world scenario of boosting waning immunity of general population receiving WT mRNA vaccines^10,11^, we sought to investigate the effect of homologous boosting with WT LNP-mRNA or heterologous boosting with BA.1 LNP-mRNA on waning immunity of WT-vaccinated animals against Omicron BA.2. The overall antibody titer changes over time in matched booster groups showed similar trend within BA.1 and BA.2 ELISA datasets as well as within receptor-binding domain (RBD) and ectodomain (ECD) datasets (Fig. 1e). An over 18-fold time-dependent decrease in antibody titer was observed over 3 months (day 35 vs >4 months) in both BA.1 and BA.2 datasets, suggesting evident and comparable waning immunity for the two Omicron sublineages. When comparing the boosting effect of WT and BA.1 LNP-mRNAs, BA.1 LNP-mRNA consistently showed a better performance than WT in BA.1 and BA.2 datasets. The ECD-binding antibody titer increases by BA.1 LNP-mRNA were 357-fold (fold change = titer ratio – 1) and 246-fold for BA.1 and BA.2 antigens respectively, while the ones mediated by WT LNP-mRNA were 25-fold and 27-fold. The RBD dataset showed a matching, well-correlated titer pattern with ECD dataset, although its overall titer level was lower due to limited epitopes and monomer state (Fig. 1e; Supplementary Fig. S3). Compared to BA.1 antigen, both WT and BA.1 LNP-mRNAs showed weaker boosting effects on BA.2 antigen and this effect reduction was more apparent for BA.1 LNP-mRNA than WT LNP-mRNA. As the post-booster titers against BA.1 and BA.2 were quite similar, this reduction was mainly due to higher pre-booster titers against BA.2 antigen, although such pre-booster titer difference between BA.1 and BA.2 did not reach statistical significance. The data from pseudovirus neutralization assay of BA.2 correlated well with corresponding ELISA data and strengthened the forementioned findings in ELISA (Supplementary Fig. S4). The neutralizing titer enhancement mediated by WT and BA.1 boosters were 17-fold (*P* < 0.001) and 63-fold (*P* < 0.0001), respectively (Fig. 1f). Importantly, the heterotypic vaccination by Omicron BA.1 LNP-mRNA vaccine booster is more efficient at boosting neutralizing titers than WT LNP-mRNA booster (comparing boosting effect of WT vs BA.1, 64/18 = 3.6, Fig. 1f). The Omicron BA.2.12.1 subvariant is rapidly rising across the world. It gained L452Q and S704L mutations from its predecessor BA.2 subvariant. As we previously showed that mutations at L452 site have the highest individual impact on decreasing neutralization titers^12^, we went on to ask how efficient WT and BA.1 LNP-mRNA boosters are to elicit neutralizing antibodies against BA.2.12.1. To answer this question, we assessed sera neutralization activity of BA.2.12.1 infectious virus using a 50% plaque-reduction neutralization (PRNT50) assay. This assay showed a significant neutralizing titer increase (8-fold) by BA.1 booster, but not by WT booster, indicative of attenuation of BA.2.12.1 infection mediated by BA.1 booster as well as an apparent immune evasion of BA.2.12.1 from WT elicited immunity (Fig. 1g). These data highlight the benefit of receiving booster shots and advantage of BA.1-specific booster over WT booster against Omicron BA.2 as well as BA.2.12.1.

In summary, our data showed a significant drop of antibody titers over time and clear benefit of WT and BA.1 LNP-mRNA boosters on Omicron BA.1, BA.2 and BA.2.121, which justify and necessitate the use of homologous WT or heterologous BA.1 boosters in order to curb the fast spread of Omicron sublineages. The remarkable antigenic evolution of emerging variants from WT virus renders many existing clinical antibodies and vaccines lost of efficacy, which is especially evident for Omicron BA.2 and BA.2.12.1. To prevent this ever-evolving enemy breaking through our line of defense, we generated and characterized a number of variant-specific LNP-mRNAs, including B.1.351, B.1.617, and BA.1. Because of shared mutations with BA.1, BA.2 or BA.2.12.1, these variant-specific LNP-mRNAs displayed better performance of inducing neutralizing antibodies than WT LNP-mRNA in booster and non-booster settings. Characterization of these variant-specific LNP-mRNAs would pave way for developing new mRNA vaccines targeting the evolving variants.

## Supplementary information

Supplementary Information

Supplementary Dataset S1
