# Supplementary material for: Heterotypic vaccination responses against SARS-CoV-2 Omicron BA.2
Source: Cell Discov. 2022 Jul 19;8:69. doi: 10.1038/s41421-022-00435-w (PMC9295082; doi:10.1038/s41421-022-00435-w)
Supplement: Supplementary file 1 — Supplementary Information [file 41421_2022_435_MOESM1_ESM.pdf]

Supplementary information

Fang et al.

Heterotypic vaccination responses against SARS-CoV-2 Omicron BA.2

Inventory of supporting information

Supplementary figures and legends

Supplementary source data and statistics

Provided in excel file, “20220528_data_summary.xlsx”.

Supplementary figures and legends

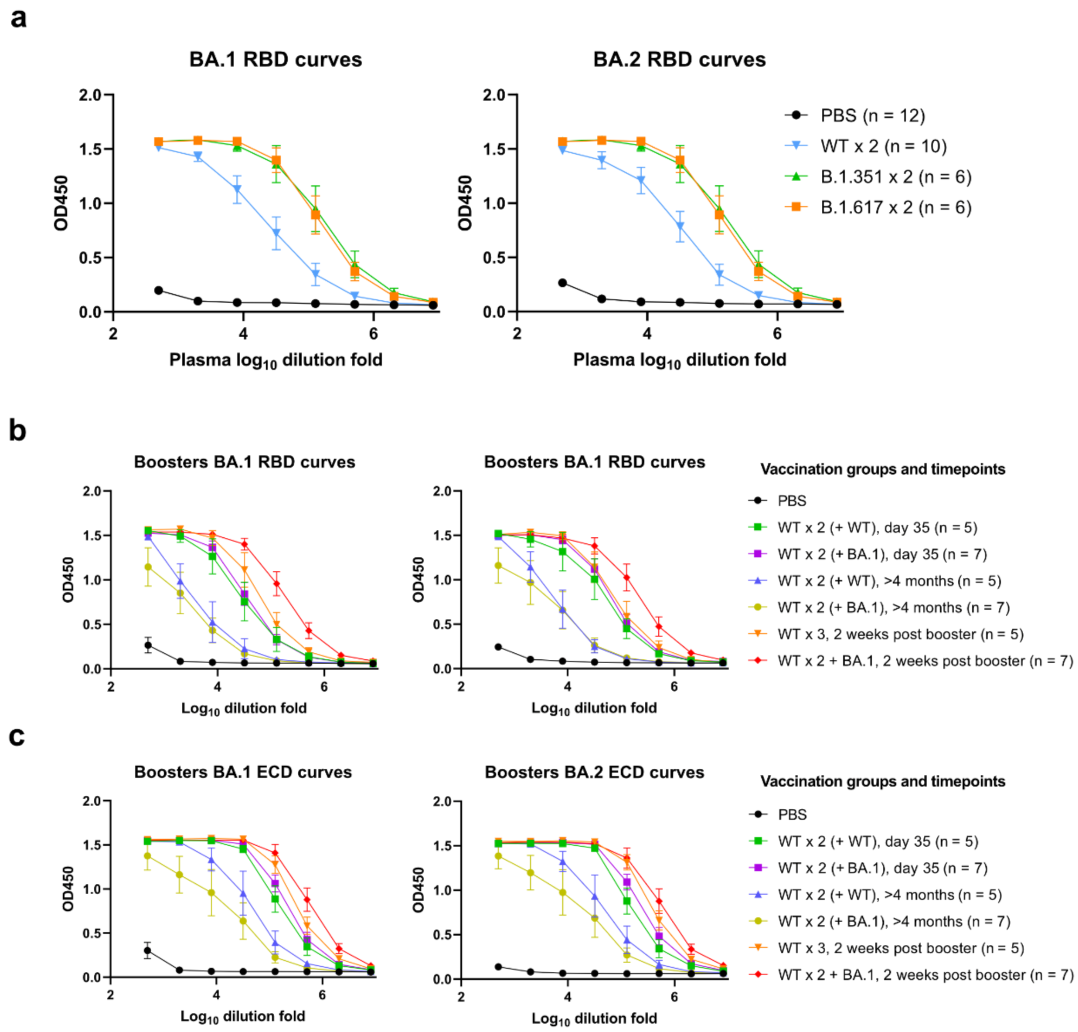

Supplementary Figure S1. ELISA dose-response curves of serially diluted plasma or sera collected at indicated time points from mice vaccinated with WT or variant specific LNP-mRNA.

a, Titration curves against BA.1 (left) and BA.2 (right) RBDs by samples from mice immunized with two doses of 1ug WT, B.1.351 or B.1.617 LNP-mRNAs.

b, Titration curves against BA.1 (left) and BA.2 (right) RBDs by mice samples before and after 10ug WT or BA.1 LNP-mRNA booster shots.

c, Titration curves against BA.1 (left) and BA.2 (right) ECDs by mice samples before and after 10ug WT or BA.1 LNP-mRNA booster shots.

The average OD450 response were shown as mean ± s.e.m. and plotted against serial $\log_{10}$-transformed sample dilution points.

a

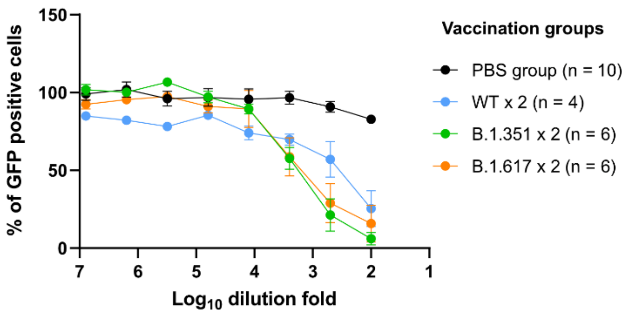

b

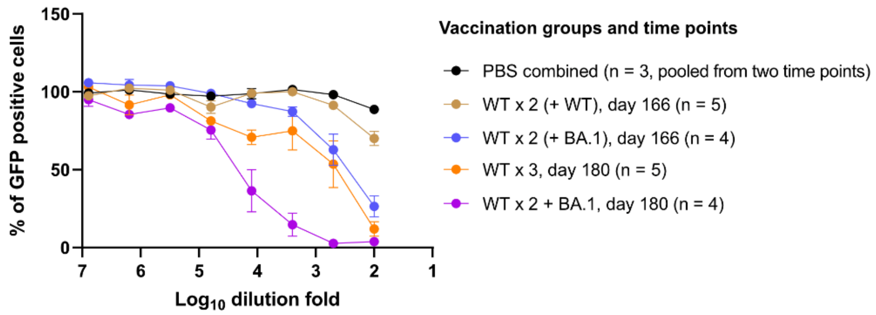

c

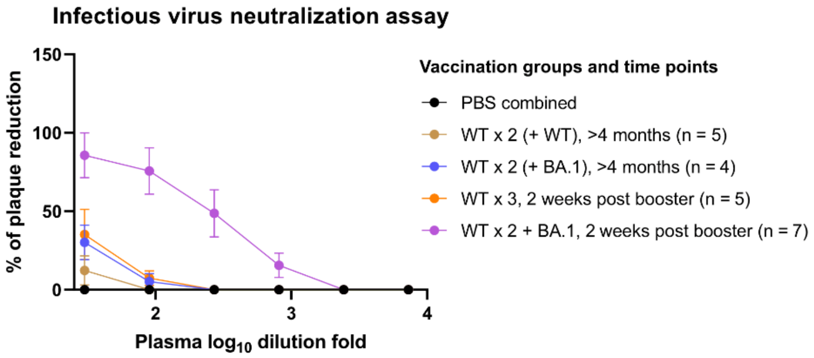

Supplementary Figure S2. Neutralization titration curves of serially diluted plasma or sera collected at indicated time points from mice vaccinated with WT or variant specific LNP-mRNA.

a, Neutralization curves of BA.2 pseudovirus by samples from mice immunized with two doses of 1ug WT, B.1.351 or B.1.617 LNP-mRNAs.

b, Neutralization curves of BA.2 pseudovirus by samples before and after 10ug WT or BA.1 LNP-mRNA booster shots.

c, Neutralization curves of BA.2.12.1 infectious virus by samples before and after 10ug WT or BA.1 LNP-mRNA booster shots.

The average GFP positive rates or pseudovirus infection rates were shown as mean ± s.e.m. and plotted against serial log_{10}-transformed sample dilution points. A subset of PBS and WT (with and without boosters) group mice were characterized in pseudovirus and infectious virus neutralization assay due to depletion of collected samples.

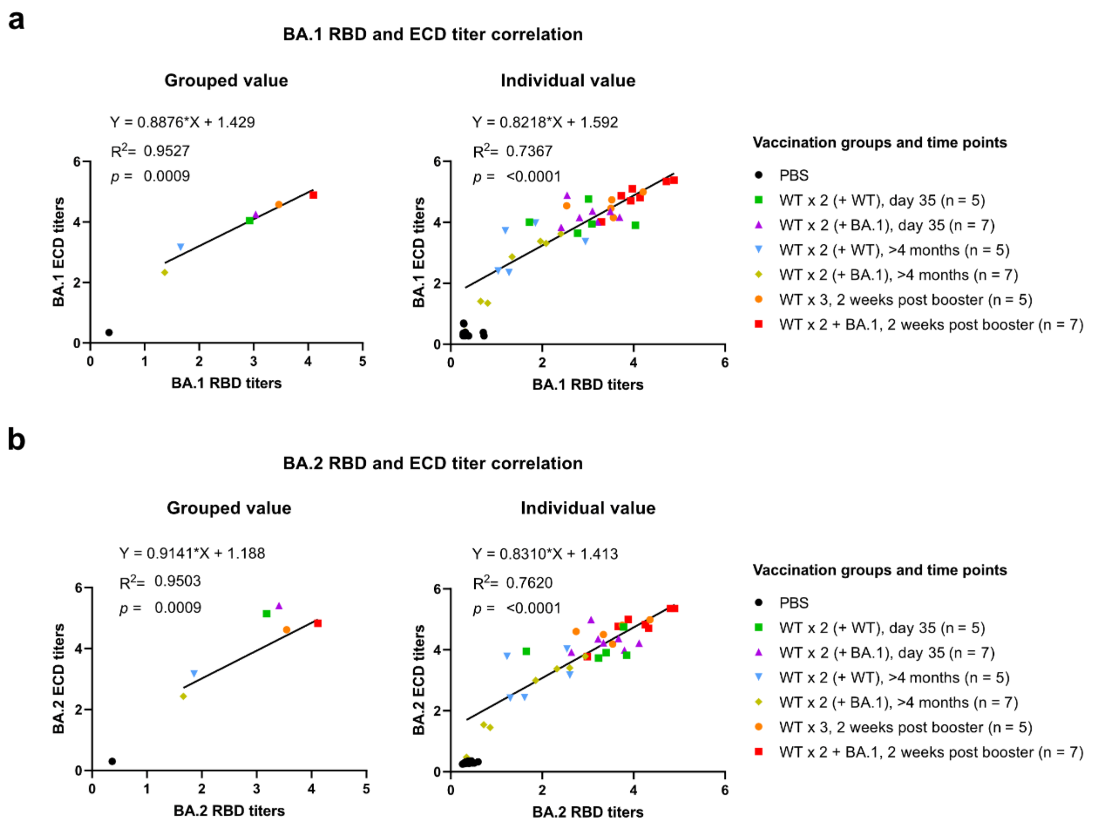

Supplementary Figure S3. Correlation between antibody titers measured by RBD and ECD of BA.1 and BA.2 in ELISA.

Antibody titers quantified using ECD of BA.1 (a) or BA.2 (b) were shown on y axis as $\log_{10}$ AUC and plotted against RBD binding antibody titers on x axis ($\log_{10}$ AUC). Titer values were either from mean of matched vaccination group (left) or individual animal (right).

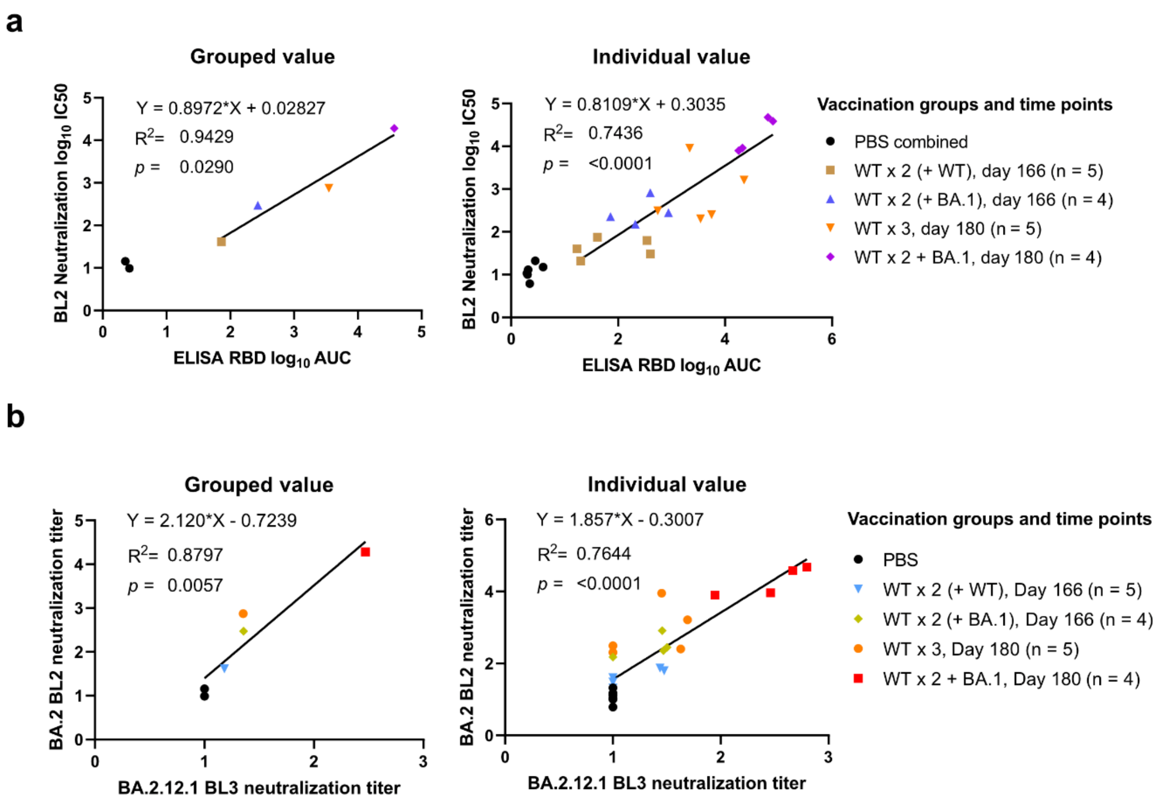

Supplementary Figure S4. Correlation between antibody titers measured by ELISA, pseudovirus (BL2) and infectious virus (BL3) neutralization assay.

Pseudovirus neutralizing antibody titers were shown on y axis as log_{10} reciprocal IC50 and plotted against ELISA binding antibody titers (log_{10} AUC, a) or B.2.12.1 infectious virus neutralizing titers (Log_{10} IC50, b) on x axis. Titer values were either from mean of matched vaccination group (left) or individual animals (right).

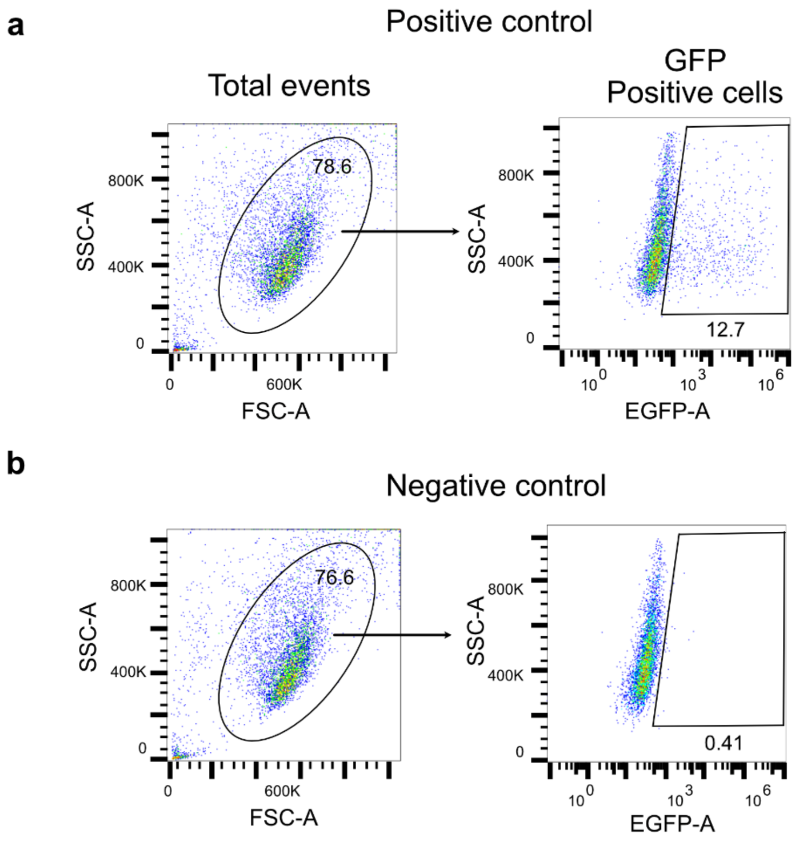

Supplementary Figure S5. Representative flow cytometry gating strategy used in pseudovirus neuralization assay for detecting GFP positive or infected cells.

Methods

Molecular cloning and mRNA transcription

The coding sequence of Omicron BA.2 spike were derived from isolates in GISAID EpiCoV database (EPI_ISL_6795834.2). The spike plasmids were linearized by restriction enzymes and transcribed to mRNA by in vitro T7 RNA polymerase (NEB, Cat # E2060S) as previously described[1,2].

Cell culture

293T and hACE2-293FT cells were maintained in Dulbecco's minimal essential medium (DMEM, Fisher) supplemented with 10% fetal bovine serum (Hyclone) and penicillin (100 U/ml)-streptomycin (100 ug/ml). Cells were split ever other day at a 1:4 ratio when confluency is over 90%.

Lipid nanoparticle mRNA preparation

The lipid nanoparticle mRNA were prepared as previously described[1,2]. In brief, lipid mixture was dissolved in ethanol and mixed with mRNA in pH 5.2 sodium acetate. The mRNA encapsulated by LNP (LNP-mRNA) was then exchanged to PBS using 100kDa Amicon filter (Macrosep Centrifugal Devices 100K, 89131-992). The DLS device was used to validate the size distribution of LNP-mRNA (DynaPro NanoStar, Wyatt, WDPN-06). The encapsulation rate and mRNA amount were determined by Quant-iT™ RiboGreen™ RNA Assay (Thermo Fisher).

Animal vaccination

Animal immunization were performed previously on 6-8 weeks female C57BL/6Ncr mice purchased from Charles River in two sets of experiments: 1) sequential vaccination with two doses of 1µg WT LNP-mRNA followed by 10µg Omicron BA.1 or WT boosters[2] ; 2) vaccination with two doses of 1µg WT, B.1.351, B.1.617 LNP-mRNA[1]. Retro-orbital blood were collected two weeks post boost (2nd dose, day 35), right before boosters (day 127 or 166 in two independent

experiments, batch 1 and batch 2), and two weeks post boosters (3rd dose, day 140 in batch 1 and day 180 in batch 2).

ELISA and Pseudovirus neutralization assay

The binding and neutralizing antibody titers were determined by ELISA and pseudovirus neutralization assay as previously described[1,2]. The Omicron BA.1 RBD and BA.2 RBD used in ELISA were purchased from Sino Biological (Cat. No. 40592-V08H121) and AcroBiosystems (Cat. No. SPD-C522g-100ug) respectively. The Omicron BA.1 ECD and BA.2 ECD used in ELISA were purchased from Acrobiosystems (BA.1 ECD, SPN-C52HZ and BA.2 ECD, SPN-C5223) respectively. The pseudovirus plasmids were generated based on the WT plasmid which was a gift from Dr. Bieniasz's lab[3].

Infectious virus neutralization assay

Sera from immunized mice were heat treated for 30 min at 56°C. Sixfold serially diluted plasma, from 1:30 to 1:7290 were incubated with 10^3 BA.2.12.1 SARS-CoV-2 variant, for 1 h at 37 °C. The mixture was subsequently incubated with TMPRSS2-VeroE6 in a 12-well plate for 1h, for adsorption. Then, cells were overlayed with MEM supplemented $NaHCO_3$, 4% FBS, 0.6% Avicel RC-581 mixture. Plaques were resolved at 40 h post infection by fixing in 10% formaldehyde for 1 h followed by staining in 0.5% crystal violet dissolved in 20% ethanol. All experiments were performed in parallel with baseline controls sera, in an established viral concentration to generate 60-120 plaques/well.

Statistics

For Figure 1, individual data points represent value from each mouse sample and are shown on dot-bar plots as mean ± s.e.m.. To assess statistical significance, two-way ANOVA with Tukey's multiple comparisons test was used. Statistical significance labels: * $p < 0.05$; ** $p < 0.01$; *** $p < 0.001$; **** $p < 0.0001$. Non significant comparisons are not shown.

Institutional approval

This study has received institutional regulatory approval. All recombinant DNA (rDNA) and biosafety work were performed under the guidelines of Yale Environment, Health and Safety (EHS) Committee with approved protocols (Chen 18-45, 20-18, 20-26). All animal work was performed under the guidelines of Yale University Institutional Animal Care and Use Committee (IACUC) with approved protocols (Chen-2020-20358; Chen 2021-20068).

Data availability

All source data and statistics are provided in this article and its supplementary table excel file (20220528_data_summary.xlsx). Additional information related to this study are available from the corresponding author upon reasonable request.

Code availability

No custom code was used in this study.

Reference

1 Lei, P., et. al. Systems immune profiling of variant-specific vaccination against SARS-CoV-2. *BioRxiv*, doi:https://doi.org/10.1101/2021.12.02.471028 (2021).

2 Fang, Z., et. al. Omicron-specific mRNA vaccination alone and as a heterologous booster against SARS-CoV-2. *BioRxiv*, doi:https://doi.org/10.1101/2022.02.14.480449 (2022).

3 Schmidt, F. *et al.* Measuring SARS-CoV-2 neutralizing antibody activity using pseudotyped and chimeric viruses. *J Exp Med* **217**, doi:10.1084/jem.20201181 (2020).
